# First Report of Response to Tarlatamab in a Patient With DLL3-Positive Pulmonary Carcinoid: Case Report

**DOI:** 10.1016/j.jtocrr.2024.100750

**Published:** 2024-10-19

**Authors:** Alissa J. Cooper, Natasha Rekhtman, Marina K. Baine, Marie C. Thomas, Alia C. Lynch, Ryan D. Gentzler

**Affiliations:** a Department of Thoracic Oncology, Memorial Sloan Kettering Cancer Center, New York, New York; b Department of Pathology and Laboratory Medicine, Memorial Sloan Kettering Cancer Center, New York, New York; c Division of Hematology/Oncology, Department of Medicine, University of Virginia Cancer Center, Charlottesville, Virginia; d Department of Pharmacy Services, UVA Health, Charlottesville, Virginia

**Keywords:** Carcinoid, Neuroendocrine, Tarlatamab, Case report

## Abstract

Tarlatamab, a DLL3-targeting bispecific T-cell engager, has rapidly assumed the role of a new standard of care in the later-line treatment of extensive-stage SCLC. Little is known about the efficacy of tarlatamab in other histologies such as DLL3-expressing metastatic pulmonary carcinoid tumor, a clinical entity without many approved management options. Here, we report the case of a patient with metastatic atypical carcinoid tumor which had progressed on multiple previous therapies. Her tumor strongly expressed DLL3 protein and had clinical response to tarlatamab therapy. This case indicates that this novel therapy may be an efficacious option in other pulmonary neuroendocrine cancers.

## Introduction

Pulmonary carcinoids are a rare subtype of lung neuroendocrine tumor, accounting for approximately 1% to 2% of lung malignancies in adults. Tumors are characterized as “typical carcinoids” (low grade) or “atypical carcinoids” (intermediate grade) based on the mitotic count and the presence or absence of necrosis. Clinical phenotype often dovetails with these histologic distinctions; typical carcinoids often display indolent growth with infrequent metastasis and atypical carcinoids may have more aggressive behavior with quicker tumor growth and dissemination. For patients diagnosed with metastatic pulmonary carcinoid tumor, there are few recommended treatment options often with low response rates.^1^, ^2^, ^3^

DLL3 expression is often observed on high-grade neuroendocrine cancers, such as SCLC, but it has also been identified in approximately 30% to 40% of pulmonary carcinoids.^4^ Thus, novel DLL3-targeting agents offer new hope for effective therapies for patients with pulmonary neuroendocrine cancers beyond SCLC. For example, obrixtamig (DLL3-targeting T-cell engager) induced an objective response in seven of 10 patients with large cell neuroendocrine carcinoma.^5^ Here, we report a case of a patient with metastatic atypical carcinoid refractory to multiple therapies. Her tumor was found to strongly express DLL3 and had an impressive clinical response to tarlatamab therapy.

## Case Presentation

A 61-year-old woman with no history of tobacco use and past medical history of hypothyroidism presented with cough and worsening dyspnea in the preceding 10 months. A computed tomography (CT) of the chest identified a 5-cm mass invading the mediastinum with an endobronchial component and an enlarged subcarinal lymph node. Endobronchial ultrasound-guided biopsy of the right mainstem endobronchial mass and subcarinal lymph node revealed a neoplasm initially interpreted as SCLC. She then came to the University of Virginia Cancer Center for further recommendations. Magnetic resonance imaging (MRI) of the brain identified an indeterminate 2-mm lesion. FDG-PET/CT imaging confirmed hypermetabolic activity in known thoracic disease and indeterminate uptake in a small left adrenal nodule. Consensus at multidisciplinary thoracic tumor board was that this most likely represented stage IV (extensive-stage) disease, and she started carboplatin, etoposide, and atezolizumab.

Given the patient’s never-smoking status, further workup was pursued and additional biopsies and debridement of right mainstem bronchus were performed by interventional pulmonology 1 day before starting systemic therapy. Pathologic result was more consistent with an atypical carcinoid, as evidenced by intermediate Ki-67 proliferative index of 30%, retained RB, and wild-type pattern of p53 expression. Serum chromogranin A level was elevated at 158. PET/dotatate revealed increased uptake in the known primary lesion and right supraclavicular lymph node without definitive evidence of uptake elsewhere. Notably, there was no abnormal uptake in the left adrenal gland. On the basis of the new stage IIIC atypical carcinoid diagnosis, need for local control, and absence of definitive evidence of metastatic disease, concurrent radiation was started with cycle #2 and atezolizumab was discontinued. CT imaging at completion of chemotherapy and radiation revealed no change in size with some evidence of right mainstem soft tissue thickening and associated symptoms of worsening cough.

She underwent repeat bronchoscopy with tumor debridement and argon plasma coagulation. Tissue from the right mainstem bronchus had residual persistent atypical carcinoid, and endobronchial ultrasound biopsy of level 7 lymph node also revealed metastatic carcinoid with Ki-67 of 16.4%. Given residual disease, she started monthly octreotide long-acting repeatable (LAR) injections. Unfortunately, PET/Dotatate imaging revealed multiple new bone metastases and a newly enlarged left axillary lymph node 4 months after starting octreotide. A left axillary lymph node was biopsied and confirmed metastasis of atypical carcinoid. A brain MRI identified 10 new metastases, all less than or equal to 1 cm.

**Figure 1:**
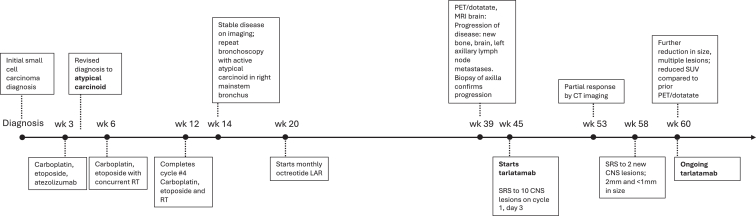
Diagnostic and treatment timeline. CNS, central nervous system; CT, computed tomography; LAR, long-acting repeatable; MRI, magnetic resonance imaging; PET, positron emission tomography; RT, radiation therapy; SRS, stereotactic radiosurgery; SUV, standard uptake value.

**Figure 2:**
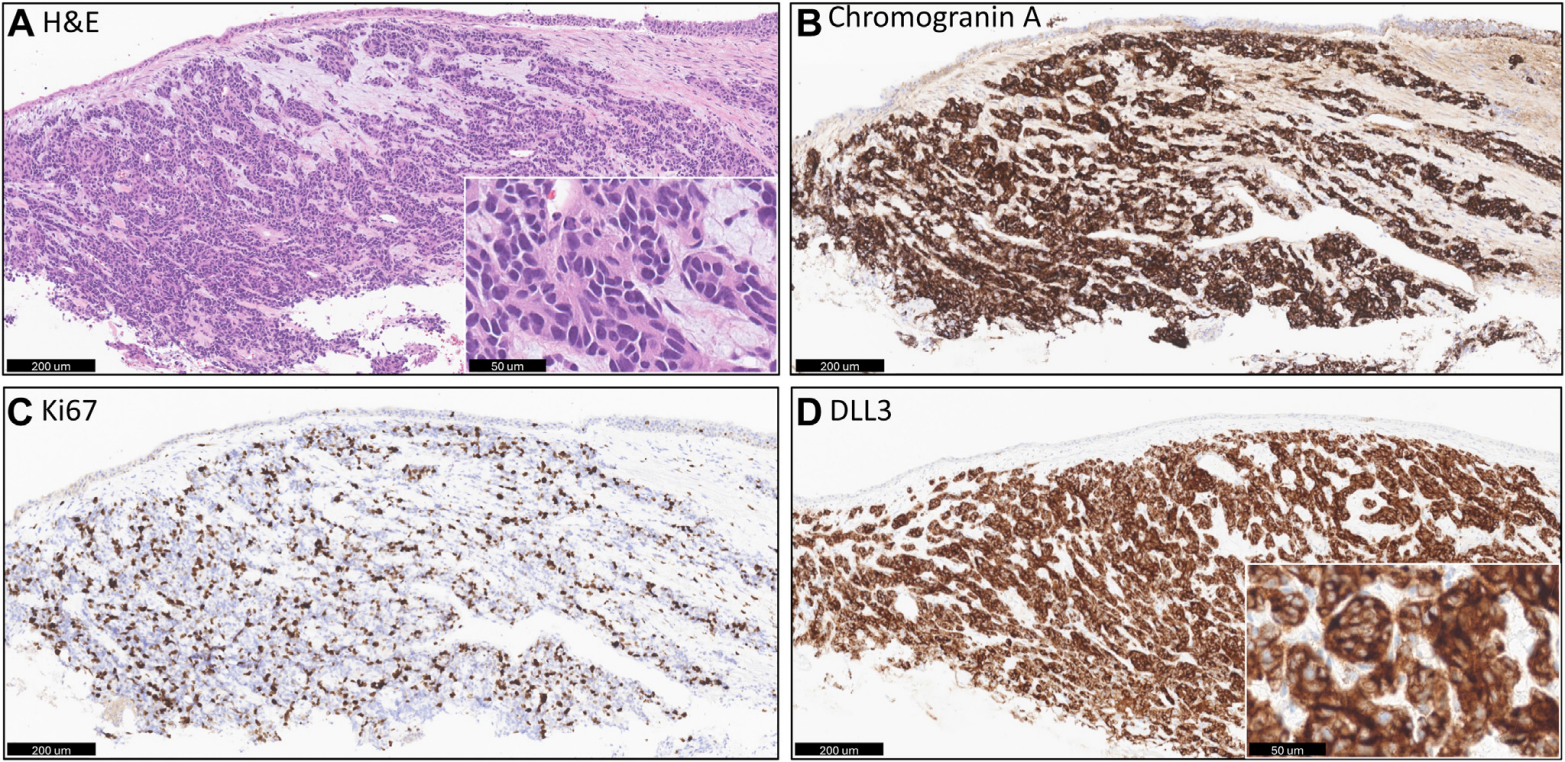
Histopathology and DLL3 expression. (*A*) Hematoxylin and eosin of the endobronchial biopsy reveals a nested tumor with moderate granular cytoplasm, minimal cytologic atypia, and neuroendocrine features. (*B*) The tumor expresses chromogranin A and (*C*) has a Ki-67 proliferative index of 30%, consistent with the diagnosis of atypical carcinoid. (*D*) DLL3 reveals strong and diffuse membranous and cytoplasmic expression.

**Figure 3:**
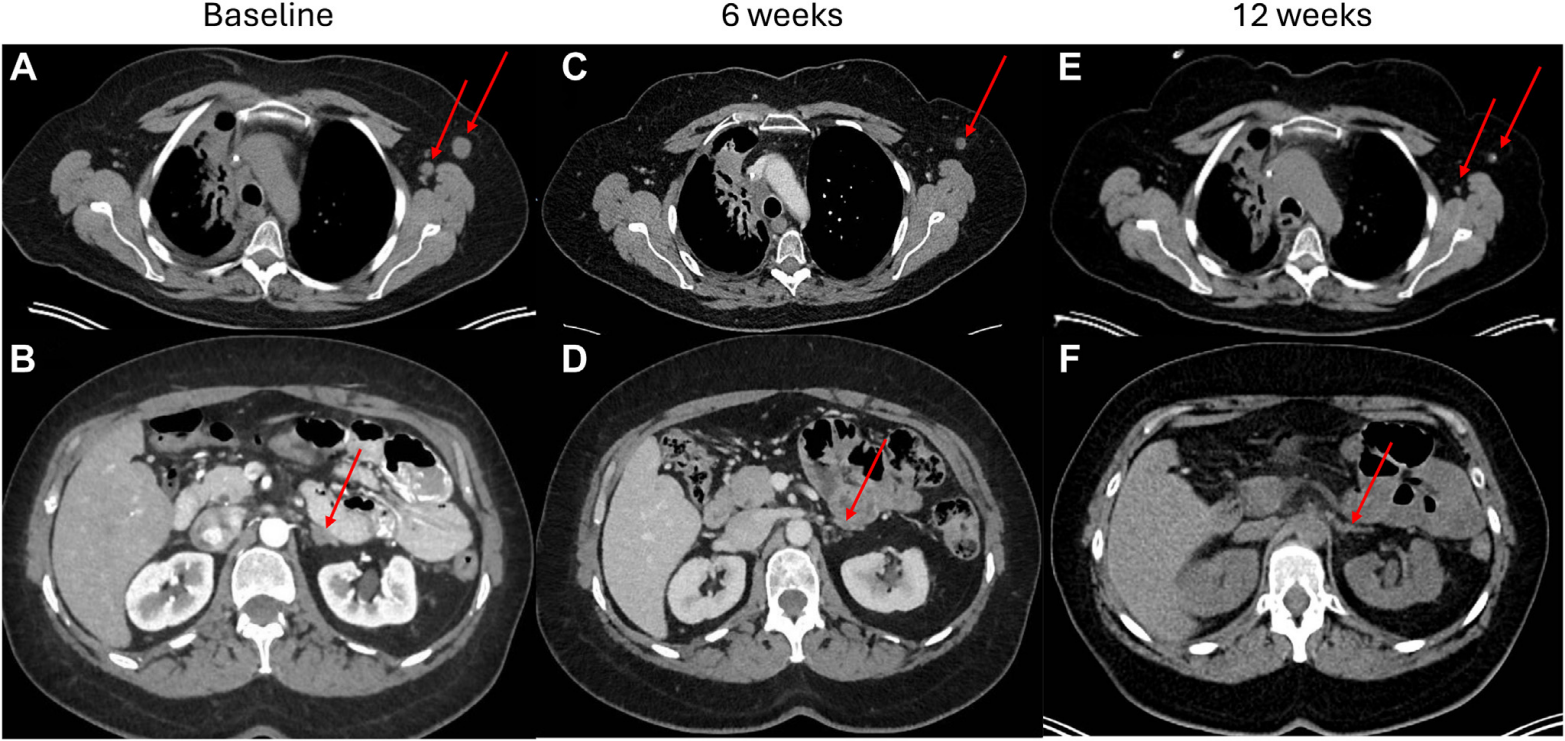
Baseline, 6-week, and 12-week imaging of key lesions revealing response. (*A*) Baseline, CT component of PET/Dotatate, enlarged left axillary lymph nodes; (*B*) baseline, CT abdomen, left adrenal metastasis; (*C*) 6 weeks, CT chest, left axillary node; (*D*) 6 weeks, CT abdomen, left adrenal metastasis; (*E*) 12 weeks, CT component of PET/Dotatate, left axillary lymph nodes; (*F*) 12 weeks, CT component of PET/Dotatate, left adrenal metastasis. CT, computed tomography; PET, positron emission tomography.

After 11 months from her initial diagnosis (Fig. 1), she started treatment with tarlatamab based on the aggressive clinical behavior of her cancer and previous literature documenting DLL3 expression in pulmonary carcinoid. DLL3 immunohistochemistry was performed after treatment was started and revealed strong expression on 100% of cells (Fig. 2, *A*–*D*). On cycle 1, day 3, her brain metastases were treated with stereotactic radiosurgery. CT imaging 6 weeks after initiation of tarlatamab revealed disease response, with reduction in left axillary lymph node from 1.5 cm to 0.9 cm and left adrenal nodule from 1.3 cm to 0.5 (Fig. 3, *A*–*D*). Decreases in the previously radiated right paratracheal mass, from 2.4 to 1.9 cm, and subcarinal lymph node, from 1.6 to 1.1 cm, were also noted. PET/Dotatate imaging at week 12 (Fig. 3*E* and *F*) was notable for further decrease in the size of right hilar mass, left adrenal nodule, and left axillary lymph nodes. The standard uptake value decreased from baseline of 6.4 to 4.7 in the right paratracheal region, 2.2 to 1.3 in the left axilla, and 2.4 to 1.2 in the left frontal calvarium bone lesion. Unfortunately, brain MRI identified two very small new lesions that were treated with a second course of stereotactic radiosurgery and continued tarlatamab. Throughout the first four cycles of tarlatamab, she had no evidence of cytokine release syndrome or immune effector cell-associated neurotoxicity syndrome. She had grade 1 fatigue during cycle 1 that resolved and a grade 1 dysgeusia that has persisted.

## Discussion

The management of patients with SCLC has been revolutionized by the approval of tarlatamab after disease progression following platinum-based chemotherapy. Given limited effective therapies available for patients with clinically aggressive neuroendocrine tumors other than SCLC, the application of this recently developed therapy for patients who may stand to benefit is warranted. Although elevated DLL3 expression level may have played a role in the activity of tarlatamab found in this case, it is unclear what level of DLL3 expression is needed to achieve clinical benefit. Case reports, such as this one, revealing promising activity (albeit of limited follow-up duration), provide justification for further investigation in a rigorously controlled manner, followed by extension of the approved indications for these therapies.

## Conclusion

We revealed that tarlatamab can have effective clinical activity in a patient with metastatic DLL3-expressing atypical carcinoid. This case highlights the need to further investigate the applications of novel therapies to rarer subsets of pulmonary neuroendocrine cancers with limited treatment options.

## CRediT Authorship Contribution Statement

**Alissa Cooper:** Conceptualization, Data curation, Project administration, Roles/Writing - original draft, Writing - review and editing.

**Natasha Rekhtman:** Data curation, Visualization, Writing - review and editing.

**Marina Baine:** Data curation, Visualization, Writing - review and editing.

**Marie Thomas:** Writing - review and editing.

**Alia Lynch:** Writing - review and editing.

**Ryan Gentzler:** Conceptualization, Data curation, Funding acquisition, Project administration, Visualization, Roles/writing - original draft, Writing - review and editing.

## Disclosure

Dr. Cooper reports the following conflicts of interest for the previous 3 years: receiving honoraria from MJH Life Sciences, Ideology Health, Intellisphere LLC, and MedStar Health, and consulting fees from 10.13039/100005564Gilead Sciences, Inc., and Regeneron. She reports receiving research funding to institution from Merck, Monte Rosa, AbbVie, Roche, and Amgen. Dr. Rekhtman reports the following conflicts of interest for the previous 3 years: receiving consulting fees from 10.13039/100004334Merck. Ms. Thomas reports the following conflicts of interest for the previous 3 years: receiving honoraria from MD Outlook. Ms. Lynch reports the following conflicts of interest for the previous 3 years: receiving honoraria from MJH Life Sciences and PrecisionAQ; consulting fees from 10.13039/501100022274Daiichi Sankyo. Dr. Gentzler reports the following conflicts of interest for the previous 3 years: receiving research funding to institution from Pfizer, Tempus, Nalo Therapeutics, Puma, Mirati, Bristol Myers Squibb, Dizal, Chugai, Amgen, AstraZeneca, Janssen, Daiichi Sankyo, Jounce Therapeutics, Takeda, Merck, Alliance Foundation, ECOG/ACRIN, NCI, Big Ten Research Consortium, Hoosier Cancer Research Network, and SWOG; receiving honoraria from Academy for Continued Healthcare Learning, Curio, OncLive, Aptitude Health, MedStar Health, Clinical Care Options, and American Society of Clinical Oncology; receiving travel support to meetings from Dava Oncology, Tempus, American Society of Clinical Oncology (ASCO), and International Association for the Study of Lung Cancer (IASLC); receiving consulting fees from 10.13039/100002429Amgen, 10.13039/100004328Genentech, 10.13039/100004325AstraZeneca, 10.13039/100009857Regeneron, Merus, Takeda, Gilead, Janssen, Mirati, and Daiichi Sankyo; and having leadership roles with Hoosier Cancer Research Network, ASCO, Journal of Clinical Oncology, NCI Investigational Drug Steering Committee, and IASLC Conference Planning Committees. Baine declares no conflicts of interest.
